# An Approach to Segment and Track-Based Pedestrian Detection from Four-Layer Laser Scanner Data

**DOI:** 10.3390/s19245450

**Published:** 2019-12-11

**Authors:** Mingfang Zhang, Rui Fu, Wendong Cheng, Li Wang, Yong Ma

**Affiliations:** 1 Beijing Key Lab of Urban Intelligent Traffic Control Technology, North China University of Technology, Beijing 100144, China; wangli939@ncut.edu.cn; 2 Key Laboratory for Automotive Transportation Safety Enhancement Technology of the Ministry of Communication, Chang’an University, Xi’an 710064, China; furui@chd.edu.cn (R.F.); chengwendong@foxmail.com (W.C.); mayong@chd.edu.cn (Y.M.)

**Keywords:** pedestrian detection, feature correlation analysis, laser scanner, track classification, probability data association filter

## Abstract

Pedestrian detection is a critical perception task for autonomous driving and intelligent vehicle, and it is challenging due to the potential variation of appearance and pose of human beings as well as the partial occlusion. In this paper, we present a novel pedestrian detection method via four-layer laser scanner. The proposed approach deals with the occlusion problem by fusing the segment classification results with past knowledge integration from tracking process. First, raw point cloud is segmented into the clusters of independent objects. Then, three types of features are proposed to capture the comprehensive cues, and 18 effective features are extracted with the combination of the univariate feature selection algorithm and feature correlation analysis process. Next, based on the segment classification at individual frame, the track classification is conducted further for consecutive frames using particle filter and probability data association filter. Experimental results demonstrate that both back-propagation neural network and Adaboost classifiers based on 18 selected features have their own advantages at the segment classification stage in terms of pedestrian detection performance and computation time, and the track classification procedure can improve the detection performance particularly for partially occluded pedestrians in comparison with the single segment classification procedure.

## 1. Introduction

Accurate and reliable obstacle classification is an important task for the environment perception module in the autonomous vehicle system, since various participants exist in the traffic environment and the motion properties of the surrounding participants directly affect the path planning of an autonomous vehicle [1]. Pedestrians are the most vulnerable traffic elements on the road, thus a great deal of attention has been paid on pedestrian detection using exteroceptive sensors. Pedestrian detection is considered as a particularly difficult problem due to the large variation of appearance and pose of human beings [2].

Most existing pedestrian detection methods rely upon several kinds of popular sensors, such as camera, radar or laser scanner [3,4]. Each sensor has its own strengths and weaknesses. The camera has been applied extensively to model the appearance characteristic of the pedestrian intuitively, but it is hard to obtain the accurate distance information and it is susceptible to illumination changes. Radar can capture the precise spatial and motion features of the obstacles, but it is not always possible to detect the static obstacles and has poor recognition capabilities. Compared with camera and radar, laser scanner enables accurate measurements and the invariance to illumination. Thus, laser scanner is used as the primary sensor in one of the most promising sensor schemes for autonomous vehicles, while camera or radar is utilized as the secondary sensor [5].

In terms of the number of scanning layers, laser scanners can be classified into three categories, namely, 2D single-layer, 2.5D multi-layer, and 3D dozens-of-layers laser scanners [6,7,8]. 2D laser scanners, e.g., the SICK LMS-111 (SICK AG, Waldkirch, Germany), provide single-layer laser beam at a fixed pitch angle, and the sparse information from single-layer point cloud is insufficient for obstacle recognition. 3D laser scanners, e.g., the Velodyne HDL-64E (Velodyne, San Francisco, CA, USA), use dozens of layers to cover 360° horizontal field of view and generate dense point cloud for omnidirectional environment modelling. In recent years, 3D laser scanners are gaining popularity in autonomous driving and intelligent vehicles, and they are usually placed on the top of a vehicle. However, the high price and external installation of 3D laser scanners limit their commercialization and popularization. Considering the practicality, 2.5D multi-layer laser scanner might be a better choice as the primary sensor than other types of laser scanners for autonomous vehicles. The existing 2.5D multi-layer laser scanner usually has four or eight layers and it is installed on the front bumper of the vehicle. Examples of 2.5D scanners include the IBEO LUX 4L and 8L (IBEO, Hamburg, Germany).

Numerous algorithms have been proposed for pedestrian detection using laser range data. Samuel et al. [7] built a pedestrian detection system based on the point cloud information from four laser planes. Particle filter was used to achieve the observation of pedestrian random movement dynamics. Carballo et al. [9] improved the pedestrian detection accuracy by introducing two novel features, namely laser intensity variation and uniformity. Gate et al. [10] used the appearance to estimate the true outlines of the tracked target. Both the geometrical and dynamical criteria of the tracked targets were utilized for pedestrian detection. Leigh et al. [11] presented a pedestrian detection and tracking system using 2D laser scanners at leg-height. Their system integrated a joint leg tracker with local occupancy grid maps to achieve robust detection. Kim et al. [12] fully exploited the feature information from 2.5D laser scanner data and developed RBFAK classifier to improve the pedestrian detection performance and reduce the computation time. Adiaviakoye et al. [13] introduced a method for detecting and tracking a crowd of pedestrians based on accumulated distribution of consecutive laser frames. This method explored the characteristics of pedestrian crowds including the velocity and trajectory. Lüy et al. [14] proposed a pedestrian detection algorithm based on a majority voting scheme using single-layer laser scanner. The scheme calculated the recognition confidence of each hypothesis over time until a high recognition confidence is achieved. Wang et al. [15] presented a framework for current frame-based pedestrian detection using 3D point clouds. A fixed-dimensional feature vector was built for each patch to solve the binary classification task. However, in their work, the precision and recall of the pedestrian detection test were unsatisfactory. Lehtomäki et al. [16] used several geometry-based point cloud features, such as local descriptor histograms, spin images, general shape and point distribution features, to improve the pedestrian detection accuracy. Xiao et al. [17] proposed a simultaneous detection and tracking method for pedestrians using 3D laser scanner. An energy function was built to incorporate the shape and motion of the point cloud, and the points belonging to pedestrians were assigned into continuous trajectories in space-time. The methods in the above literatures mainly assume that the individual people is entirely visible or the state of legs can be tracked in the classification stage. However, due to the high chance of partial occlusion, it is hard to obtain the complete contour of the individual pedestrian.

To improve the pedestrian detection performance in case of partial occlusion, some researchers attempted to use the fusion of laser and vision. García et al. [18] processed context information to enhance the pedestrian detection performance using sensor fusion of single-layer laser scanner and computer vision. Oliveira et al. [19] performed a cooperative fusion of laser and vision for pedestrian detection based on spatial relationship of parts-based classifiers via a Markov logic network. Premebida et al. [20] trained a deformable part detector using different configurations of image and laser point cloud to make a joint decision regarding whether the detected target is a pedestrian or not. It is notable that the joint calibration of a camera and laser scanner is cost-effective, and some laser points are relatively invisible to the camera.

**Figure 1:**
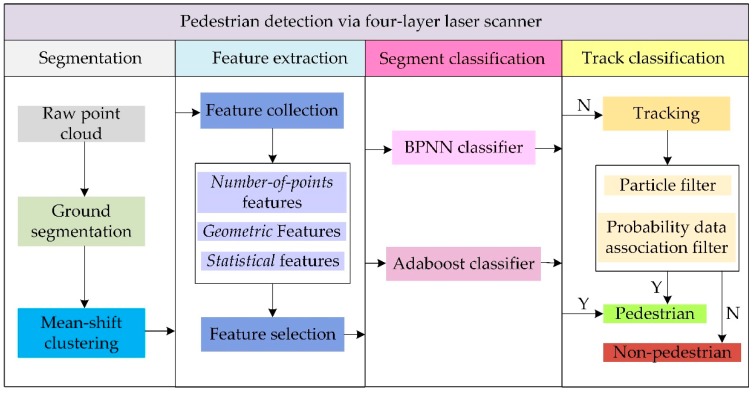
The architecture of the pedestrian detection algorithm.

Motivated by the analysis of the existing works in related literatures, a novel method for pedestrian detection using 2.5D laser scanner is presented in this paper. The laser scanner sensor adopted in this study is a four-layer laser scanner, i.e., IBEO LUX 4L, which is extensively used in Advanced Driver Assistance Systems (ADAS) and autonomous vehicles. The architecture of the proposed pedestrian detection method encompasses four components: segmentation, feature extraction, segment classification, and track classification, as shown in Figure 1. The proposed method differs from other pedestrian detection methods in two aspects. First, each layer of the raw data stream is employed to find the specific properties corresponding to objects of interest, and some new features are proposed. Most features are simple single-valued features, rather than high-level complex features. Second, in order to improve the detection accuracy when the pedestrians cross with each other and the partial occlusion exists, multi-pedestrian detection based on tracking is proposed to compensate the segment classification result.

The rest of this paper is organized as follows. The details of the proposed methods are presented in Section 2. Experimental results are analyzed and discussed in Section 3. Section 4 concludes the paper.

## 2. Proposed Methods

### 2.1. Segmentation

**Figure 2:**
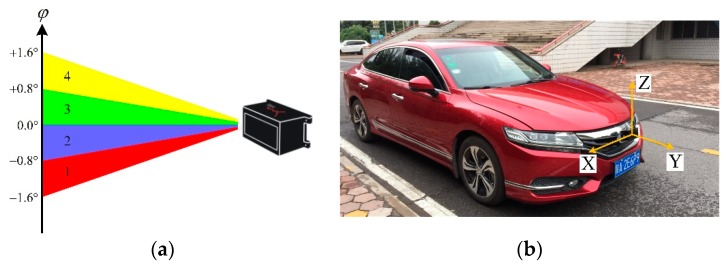
IBEO LUX 4L laser scanner. (**a**) scan layers and vertical beam divergence [22]. (**b**) the laser scanner installed on the test vehicle.

IBEO LUX 4L laser scanner is installed on the front bumper of the test vehicle, as shown in Figure 2. The raw point cloud is segmented using mean-shift clustering algorithm [21], and the clusters which are too large or too small are discarded. The point cloud of each cluster is represented by:

(1) $$P_{c}^{t}=\{p_{1}^{t},\;p_{2}^{t},\;p_{3}^{t},\;\ldots ,\;p_{N_{c}}^{t}\}$$

(2) $$p_{i}^{t}=(x_{i}^{t},\;y_{i}^{t},\;z_{i}^{t},\;l_{i}^{t})\;\mathrm{for}\;i\;=\;1,\;\ldots ,\;N_{c},\;l_{i}^{t}\in \left\{1,\;2,\;3,\;4\right\}$$

where $P_{c}^{t}$ represents a set of measurements of the *c-*th cluster collected at time *t* consisting of $N_{c}$ points; *c* is the index of the cluster; $N_{c}$ is the number of measurement points of the *c-*th cluster; $p_{i}^{t}$ denotes an individual point and each point is represented by lateral coordinate $x_{i}^{t}$, longitudinal coordinate $y_{i}^{t}$, vertical coordinate $z_{i}^{t}$, layer number $l_{i}^{t}$.

### 2.2. Feature Extraction

#### 2.2.1. Feature Collection

To characterize the local properties of the objects of interest, each cluster is divided into 4 layers and most features are computed in each of 4 layers. This subdivision method can provide a more flexible classification representation for the occluded object [23]. Apart from the previous features in literatures, some new features are proposed to develop the pedestrian cues. All features can be categorized as three types: *number-of-points-based, geometric, statistical*.

The *number-of-points-based* features are summarized in Table 1. Feature 6 is the number of the layers which has over five measurement points. The number of points change over the layers, thus the relationship between the number of points vs. each layer is utilized as the effective feature by fitting the number of points in each layer with a linear or a second-order quadratic equation. Feature 7 is the slope of the linear fit for the number of points vs. each layer. 

Features 8 and 9 are the first- and second-order coefficients of the quadratic fit for the number of points vs. each layer, respectively [10]. Feature 10 denotes the distance between the central point of the horizontal projection and the origin of the coordinate system. Feature 11 denotes the minimum distance between the horizontal projection points and the origin of the coordinate system. Features 10 and 11 tend to be stable values for the pedestrian cluster, since the region of human leg is similar, and the number of points of the pedestrian cluster decreases as the distance between the pedestrian and the laser scanner increases.

**Figure 3:**
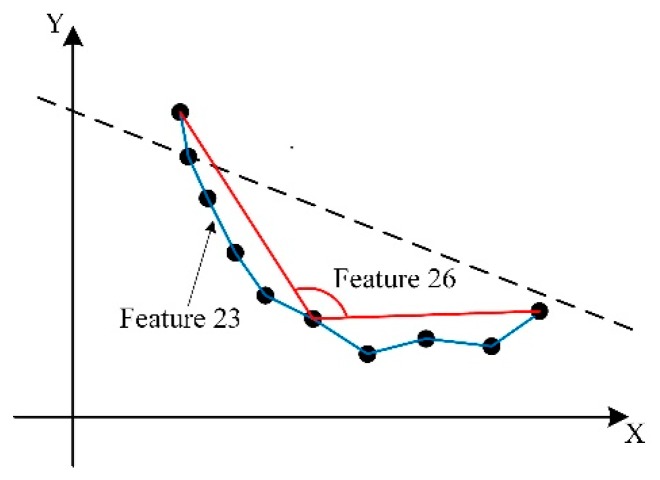
The diagram of the horizontal projection curve. The dashed line denotes the fitted line of the horizontal projection points.

The *geometric* features describe the shape of the cluster intuitively and these features are summarized in Table 2. Feature 12 from [24] is the residual sum of squares of the vertical distance between the projection points and the least-squares-fitting line which is calculated with the projection points. Feature 12 describes the proximity of the distribution of the projection points to the straight line. In the formula, *p_xy,i_* denotes the projection of the *i-th* point in the cluster on the horizontal plane, and *p_l,i_* denotes the intersection between vertical line from the point *p_xy,i_* to the fitted straight line and the fitted line. Features 13 to 15 measure the length, width and the area of the 2D rectangular bounding box of the scan points, respectively. Features 16 to 21 represent the density of the scan points in each layer. Features 23 to 29 are used to describe the distribution of the scan points on the legs as they represent the proximity of the shape of the scan cluster to the arc or ellipse. The horizontal projection of the scan points are connected in a curve with the adjacent points along the fitted line direction, as shown in Figure 3. Features 23 to 27 reflect the properties of this projection curve. Specifically, Features 23 and 25 measure the length and bend of the horizontal projection curve, respectively. Features 26 and 27 use the inscribed angle of the projection curve to represent the curvature [25]. The inscribed angle *α_i_* at each scan point refers to the angle between two lines connecting this point with two ends of the curve, as shown in Figure 3. 

The closer the mean value of inscribed angles is to 90°, the closer the curve shape is to the circle. Features 28 and 29 are defined with the least square fitting circle of the horizontal projection cluster of the scan points. Feature 28 is the sum of the residual squares of the vertical distance between the projection points and the fitting circle to imply the closeness of the projection cluster to the circle. 

The *statistical* features also provide the cues of cluster characteristics to distinguish the pedestrian from non-pedestrian, although the physical meanings of these features are not clear. The *statistical* features are summarized in Table 3. Feature 30 from [26] represents the variance of the distance between the scan points in the cluster and the centroid of the cluster in 3D coordinate system. Feature 31 represents the variance of the distance between the horizontal projection points of the cluster and the mean of the horizontal projection points. Feature 32 from [24] represents the variance of the distance between the horizontal projection points of the cluster and the median of the horizontal projection points. Features 33 to 35 from [26] are the square of the second-, third-, and fourth-order centre-spaces between the horizontal projection points of the cluster and the mean of the horizontal projection points, respectively.

**Figure 4:**
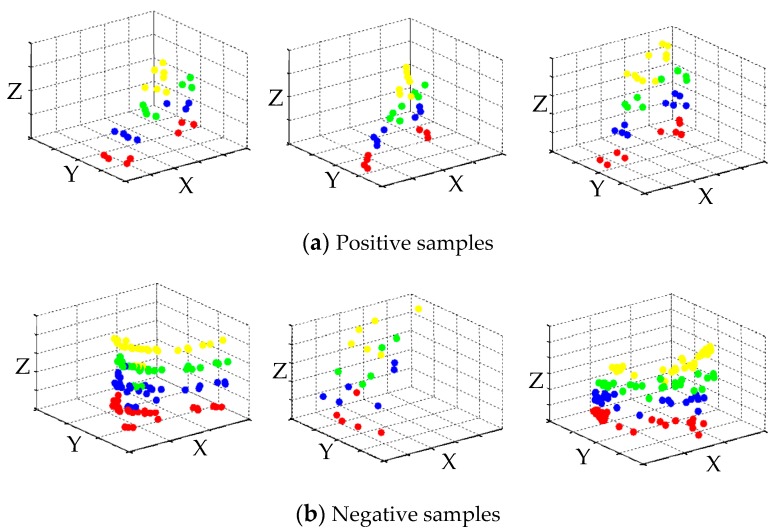
The labelled samples for pedestrian detection test.

In order to evaluate our method for pedestrian detection, we use the datasets collected from a test vehicle moving in busy street scenes. The test vehicle is equipped with multiple sensors including IBEO LUX laser scanner and two cameras. The camera images are used to manually label the cluster of point cloud. In total, 1262 positive samples and 2463 negative samples are labelled for the segmented objects. The positive samples includes the point cloud cluster of pedestrian with different contours at various ranges, and the negative samples includes various non-pedestrian objects such as the lamp post, trees, bicycles and cars, as shown in Figure 4. For each sample, a 35-dimensional feature vector is constructed with the above 35 features, and the label is assigned with either 1 (positive) or -1 (negative). A new sample set with feature vector is finally obtained.

#### 2.2.2. Feature Selection

After feature collection procedure, the univariate feature selection algorithm proposed by [27] is used to sort the classification failure rate for all features with a specific classifier and remove the features with high failure rate. Since feature redundancy may cause the overfitting result with low accuracy, the cross-feature correlation analysis is further combined with the univariate feature selection algorithm to remove redundant features. The details are introduced as follows.

##### Univariate Feature Selection Algorithm

**Figure 5:**
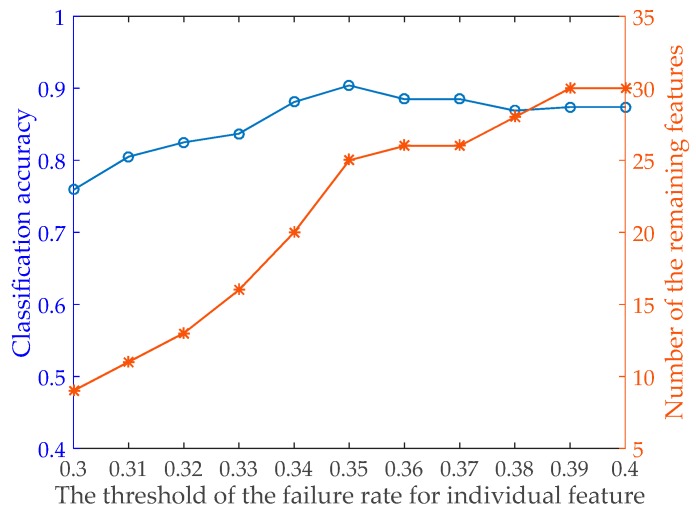
The classification performance of various feature sets based on the customized threshold of the failure rate.

The Back-Propagation Neural Network (BPNN) algorithm [28] is used as a specific classifier to select the effective features. Each feature is used separately to define an individual pedestrian classifier with BPNN algorithm and 35 classifiers are obtained in all. The 5-fold cross-validation is used to make sure the classifier is generalized over the labelled sample dataset, and the average classification failure rate of each feature classifier is shown in Table 4. Considering the features with high failure rate may make no sense to improve the pedestrian detection accuracy, we sorted the features by the classification failure rates of 35 classifiers and tested the classification performance of various feature sets based on the customized threshold *t* of the failure rate. The result is shown in Figure 5. We can see that when *t* = 35%, the number of the remaining features is 25 and the classification accuracy of the feature set including 25 remaining features is 0.904. When *t* < 35%, the number of the remaining features is less than 25 and the classification accuracy of the feature set is less than 0.904 apparently. When *t* > 35%, the number of the remaining features is larger than 25 while the classification accuracy of the feature set becomes gradually less than 0.904. Thus, we decided to define the features with the failure rate above 35% as invalid, and 25 remaining features are saved to analyze the feature correlation.

##### The Feature Correlation Analysis

Pearson correlation coefficient is used to compute the correlation between two features. It is computed by:

(3) $$r(i,j)=\frac{\mathrm{cov}(f_{i},f_{j})}{\sqrt{\mathrm{var}(f_{i})\mathrm{var}(f_{j})}}$$

where $f_{i}$ and $f_{j}$ are two feature vectors, cov is the covariance function and var is the variance function. The closer the absolute value of *r*(*i*, *j*) is to 1, the higher the correlation coefficient between two features is, the larger the possibility of the redundancy between two features is. If the absolute value of *r*(*i*, *j*) is larger than 0.9, we consider that $f_{i}$ and $f_{j}$ are redundant. After the correlation coefficients among 25 extracted features are calculated, seven groups of feature pair are demonstrated to be redundant as shown in Table 5 and Table 6. The correlation coefficients between redundant features are represented with the italics and bold font in Table 5. If two features are redundant, the feature with the higher classification error rate is removed and the other feature with the lower error rate is kept. Thus, the redundant features pairs are arranged based on the value of correlation in descending order in Table 6, and seven features with higher classification error rate are removed. According to the classification error rate in ascending order, 18 efficient features are obtained: Feature 34, Feature 1, Feature 15, Feature 16, Feature 10, Feature 32, Feature 27, Feature 5, Feature 21, Feature 25, Feature 14, Feature 26, Feature 30, Feature 19, Feature 9, Feature 20, Feature 17 and Feature 2.

### 2.3. Segment Classification

In order to select the proper classifier for pedestrian segment at individual frame, we need to evaluate the effectiveness of the segment classification algorithm and answer the following questions:

(1) What is the result of the proposed algorithm?
(2) What is the time efficiency of the proposed algorithm?
(3) Whether is the feature selection step effective?
(4) Which samples are classified wrongly? Why?

In this section, BPNN classifiers are built for pedestrian detection based on the original 35 features, 25 features from univariate feature selection procedure and 18 features from the feature correlation analysis procedure, respectively. Adaboost classifier [29] is also employed based on these feature sets to compare the performance of different classifiers comprehensively. The training and validation dataset are the same as the dataset in the univariate feature selection procedure.

### 2.4. Track Classification

#### 2.4.1. Tracking Process

**Figure 6:**
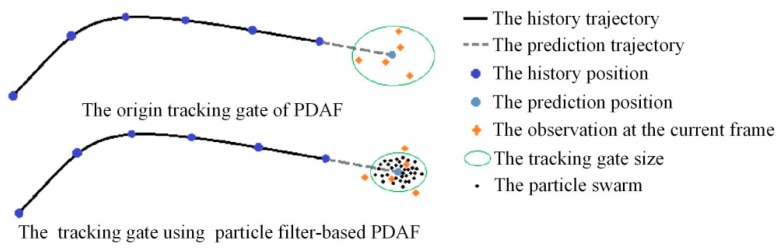
The tracking gate size of PDAF algorithm varies adaptively using particle filter.

**Figure 7:**
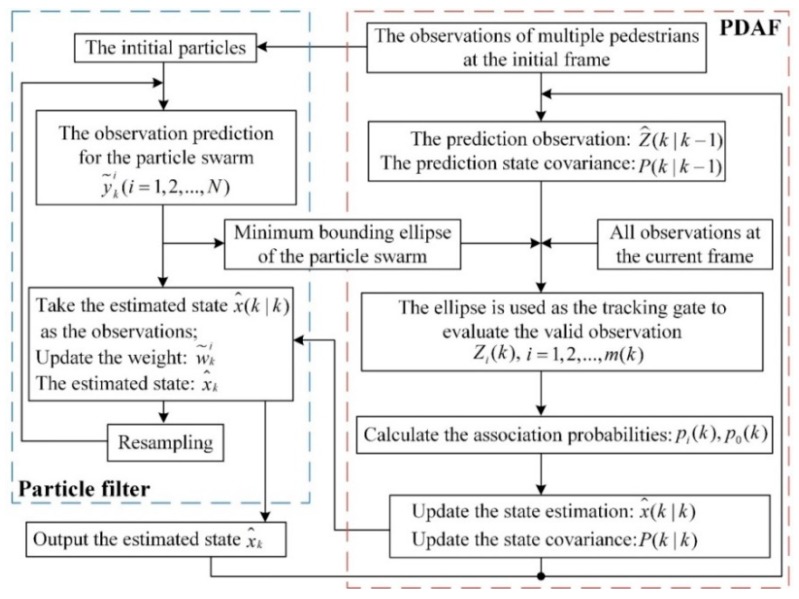
The tracking algorithm scheme based on particle filter and PDAF.

In the dynamic process of pedestrian detection, the measurement noise of laser scanner and the point cloud variation of the pedestrian affects the performance of pedestrian detection. Pedestrian tracking can improve the accuracy of pedestrian detection with the motion information and overcome the missed classification caused by partial occlusion. Probability Data Association Filter (PDAF) [30] is a frequently-used tracking algorithm due to easy-to-use and real-time performance, but it has two disadvantages. First, the tracking gate size cannot change adaptively. Second, if two trajectories intersect or they are too close, the false association will occur and cause the trajectory of the tracked target shift or overlap. In order to overcome these disadvantages, we proposed the particle filter-based PDAF algorithm for multi-pedestrian tracking. On one hand, the nonlinear motion characteristics of particle filter satisfy the variable and random motion state of pedestrian [31]. On the other hand, the propagation range of particles in particle filter algorithm can be used to adjust the tracking gate size of PDAF adaptively to match the prediction and measurement of the track, as shown in Figure 6. The global scheme of the tracking algorithm based on particle filter and PDAF is shown in Figure 7.

The steps of the proposed tracking algorithm are as follows:

*Step* *1.* Initialize the particle swarm. The particle swarms for each target are the random points within a certain circular space which is centered on this target.
*Step* *2.* The prediction observation $\hat{Z}(k|k-1)$ and covariance P(k|k-1) for each target are calculated using state equation and observation equation. The observation prediction for each particle swarm is computed at the same time.
*Step* *3.* The minimum bounding ellipse of the prediction particle swarm is calculated and the ellipse is used as the tracking gate threshold of PDAF to determine the valid observations $Z_{i}\left(k\right),i=1,2\ldots ,m(k)$ in the tracking gate. Then the associated probability between the effective observations and the target is calculated. The state estimation $\hat{x}(k|k-1)$ and covariance P(k|k) of the target is updated based on the associated probability.
*Step* *4.* The state estimation $\hat{x}(k|k-1)$ of PDAF at the current frame is taken as the observation value of the particle filter. The weight ${\tilde{w}}_{k}^{i}$ of the particle swarm and the target state estimation ${\hat{x}}_{k}$ are updated.
*Step* *5.* Particle resampling. The estimated state ${\hat{x}}_{k}$ in the particle filter is used as the final state at the current frame and returned to the PDAF. Perform *Step 2* for the next frame.

#### 2.4.2. Track Classification

Normally the detected object is determined directly as a pedestrian if it is classified as pedestrian at the current frame in the segment classification stage. When the tracked object is classified as non-pedestrian at the current frame in the segment classification stage, the classification result of the track at the last frame is used as a measure of whether the detected object is a human. At the same time, the velocity range of the track is also taken into consideration and it need satisfy the velocity constraint of the normal human. The mean velocity of the track at the last 3 frames can be obtained through the tracking process, and the velocity threshold for normal walking or running of most human beings is determined experimentally. 

## 3. Experiments and Results

### 3.1. Segment Classification Results

**Figure 8:**
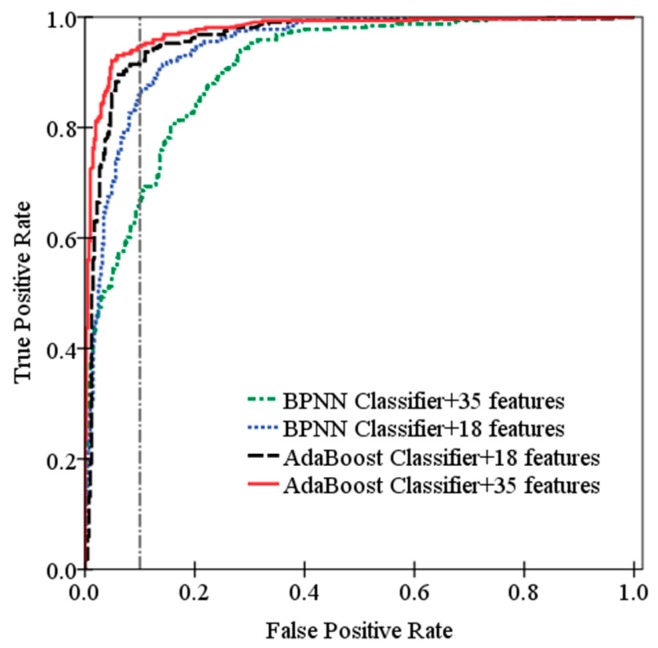
ROC curves for pedestrian detection.

To test the effectiveness of each classifier with different feature sets, Receiver Operating Characteristic (ROC) curves for each classifier are shown in Figure 8, and the results are summarized in Table 7 in terms of the accuracy, the AUC (area under the ROC) and the computation time the classifier takes for every 100 samples. The larger AUC is, the better the classification performance is. In Table 7, the accuracy of AdaBoost classifier based on 35 features reaches the highest up to 94.5%. It proves that AdaBoost algorithm does not cause overfitting with the increasing features, and 35 features we proposed are effective for pedestrian detection based on four-layer laser scanner. The accuracy of BPNN based on 35 features is the lowest, only 81.6%. After the feature selection procedure removes 17 features with high error rate and redundancy, the accuracy of BPNN based on 18 features increases to 90.7% significantly, which is close to accuracy of AdaBoost classifier. It shows that BPNN classifier is sensitive to the redundant features, and BPNN classifier based on the remaining 18 features can still obtain high accuracy and the removed features at the feature selection step contain little valid information. Overall, although the accuracy of AdaBoost classifier is higher than BPNN classifier, AdaBoost classifier takes more computation time. Note that the accuracy of AdaBoost classifier based on 35 features is only 1.3% higher than AdaBoost classifier based on 18 features, while the computation time increases by 53%. Thus, in terms of the segment classification performance and computation time, AdaBoost and BPNN classifier based on 18 features have their own advantages for pedestrian detection using multi-layer laser scanner.

**Figure 9:**
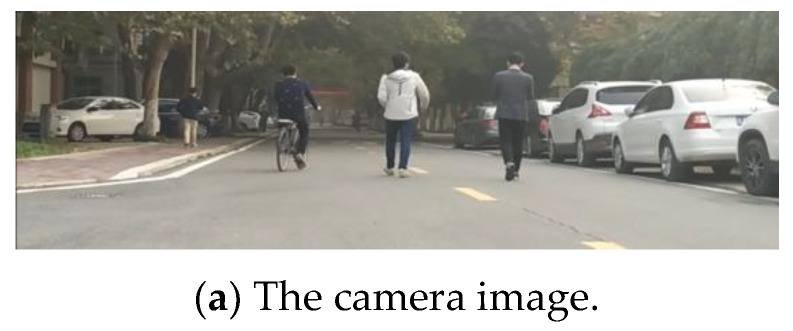

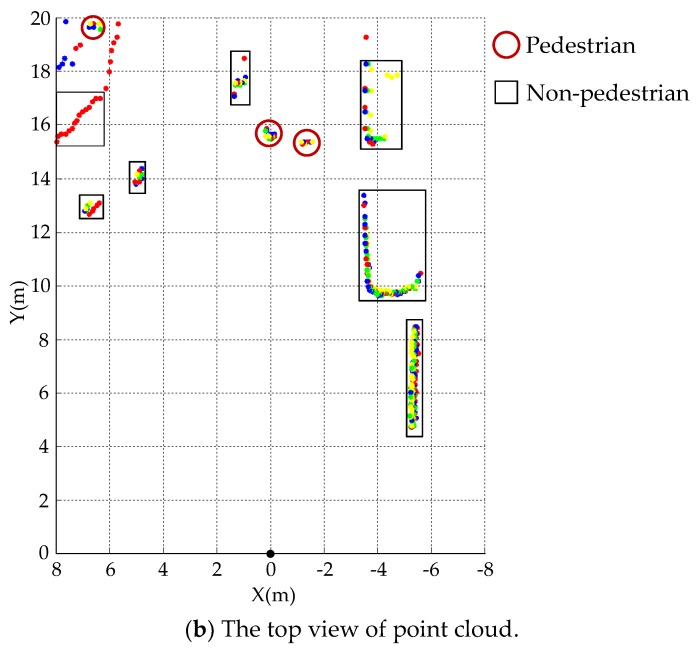
Pedestrian detection result in Scene 1.

**Figure 10:**
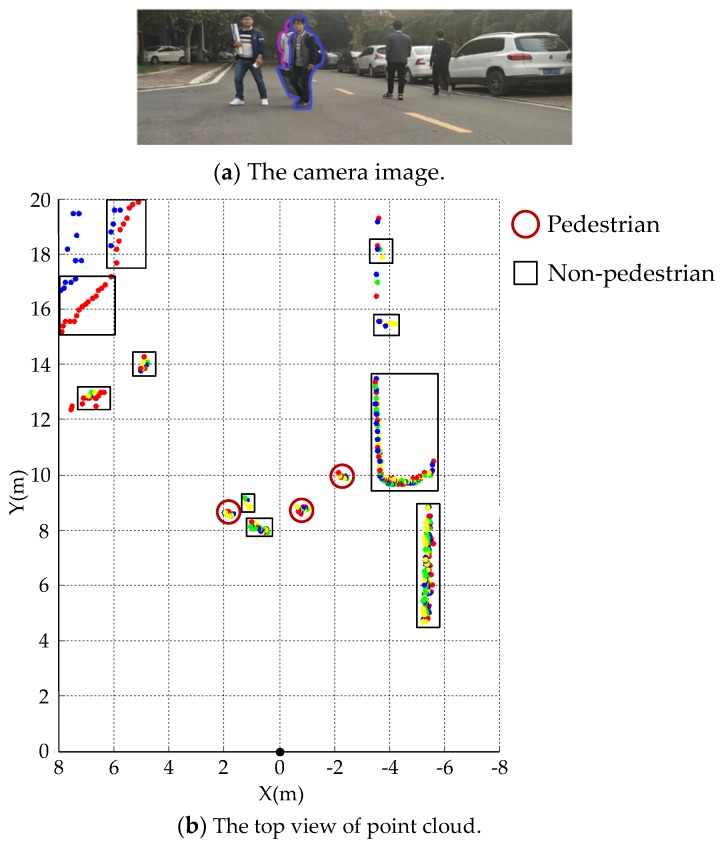
Pedestrian detection result in Scene 2.

The above analysis shows that the pedestrians can be classified correctly with AdaBoost and BPNN classifier based on 18 features in most cases. In the real road experiment, both pedestrians and non-pedestrians in the scene are correctly distinguished by BPNN classifier, as shown in Figure 9. In this figure, pedestrians are denoted by red circles, and non-pedestrians are depicted by black rectangles. After comparing the scene image with the detection results of the point cloud cluster, we find that most failure cases are caused by long range and the occlusion. As shown in Figure 10, the pedestrian surrounded by the blue curves in the image is wrongly identified in the point cloud view, since this pedestrian is occluded by the one surrounded by red curves. The occlusion, which often happens in real traffic scene, decreases the performance of the pedestrian classifier and eventually leads to misclassification. Pedestrians walking with sundries may also cause false detection. In Figure 10, the pedestrian surrounded by the blue curves in the image carried a carton, which is flush with his legs and interferes the laser scan on his legs, so that the point cloud returned from this pedestrian’s legs is abnormal in the point cloud top-view, and this pedestrian is wrongly classified as non-pedestrian. 

### 3.2. Track Classification Results

To demonstrate the performance of the proposed track classification algorithm under the influence of the occlusion and the cross of pedestrian motion trajectories, real road experiments were further carried out. The parameters of the proposed algorithm were debugged to the optimal value through a large number of tests. The initial range of the particle swarm is set as a circle with a radius of 1.39 meters centred on the pedestrian’s initial position, and the number of particles in the particle swarm is 45. The trajectory similarity threshold in trajectory-cross management is 0.35 and the number of multi-target tracker is 10.

**Figure 11:**
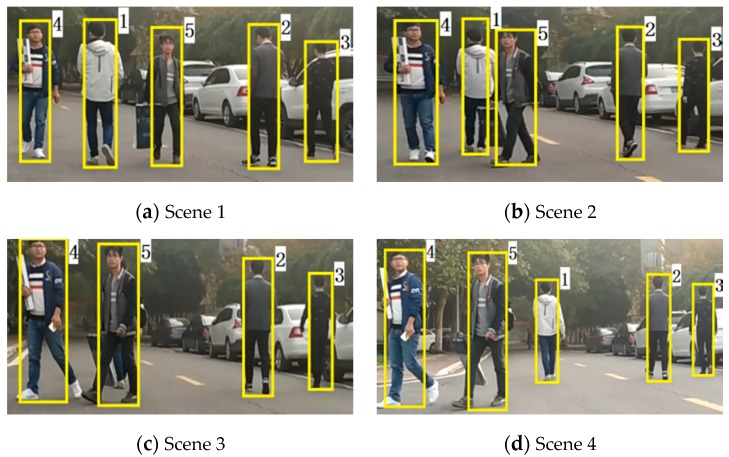
The tracking test scenario for multiple pedestrians.

As shown in Figure 11, the pedestrians in the test scenario are numbered 1 to 5, Pedestrians 1–3 gradually walked away while Pedestrians 4 and 5 were coming. It is clear that Pedestrian 1 and 5 were very close during the walking process and their trajectories crossed. The temporary occlusion leads to the pedestrian recognition failure as described in Section 2.4.1.

**Figure 12:**
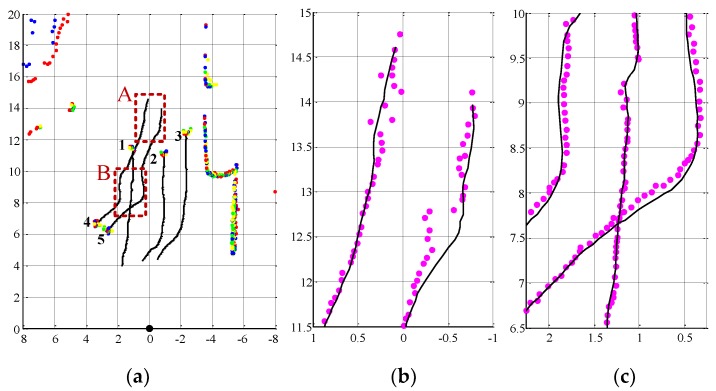
The results of tracking trajectories. (**a**) the tracking trajectories of multiple pedestrians in the point cloud scene. (**b**) the tracking trajectories in the enlarged local area A. (**c**) the tracking trajectories in the enlarged local area B.

The tracking trajectories of five pedestrians are shown in Figure 12. Figure 12a shows the distribution of the point cloud at the last frame and each pedestrian’s “dragging tail” is the tracking trajectory for 100 frames. It can be seen that all the tracking trajectories are continuous and smooth, and the proposed tracking algorithm can still accurately track multiple pedestrians, even if the temporary occlusion occurs for several frames.

Figure 12b,c enlarge the local areas of the tracking trajectory in Figure 12a to show the details, and the observations of the pedestrian positions from the pedestrian detection algorithm are denoted by the pink dots (Take the mean values of the horizontal and vertical coordinates of the pedestrian point cloud cluster as the position observation). Figure 12b shows a part of tracking trajectories of Pedestrians 4 and 5 at the tracking start time. It can be seen that the pedestrian recognition algorithm works well and the traces are continuous. As Pedestrians 4 and 5 become occluded by Pedestrian 2, the shape of the point cloud cluster dramatically changes, and the observations of the pedestrian position fluctuate greatly. However, the position fluctuations of Pedestrians 4 and 5 in the trajectory curve are significantly removed by the tracking algorithm. It means that the proposed tracking algorithm, which uses the propagation range of particle filter to adaptively determine the tracking gate for PDA, can enhance the accuracy and reliability of pedestrian position estimations.

Figure 12c shows a part of the trajectories when Pedestrians 1 and 5 intersects. We can see that Pedestrian 1 is not classified by the pedestrian recognition algorithm during some frames and the traces of the recognition results are interrupted due to the occlusion of Pedestrian 5 (corresponding to Scene 3 in Figure 11). However, the pedestrian tracking trajectory has never stopped. Thus the trajectory-cross management procedure is effective and it can overcome the false detection problem caused by the occlusion. When Pedestrians 1 and 5 are so close that their trajectories cross, the data association result and the tracking trajectories are still correct. Therefore, the trajectory-cross management method based on the trajectory similarity is effective to solve the trajectory aggregation and migration in the PDAF tracking method.

To evaluate the performance of the track classification method quantitatively, we collected a dataset including 1000 pedestrian tracks and 2000 non-pedestrian tracks. The tracks are the temporal series of the labelled point cloud segments. Each track has at least eight segments to ensure that the tracked object is observed for at least one second. 250 inconsistent tracks caused by the occlusions exist in the dataset. The dataset is divided into training and test sets at random. The pedestrian detection result is shown in Table 8. We can see that the track classification method based on BPNN or Adaboost classifier at successive frames achieves better performance than the individual segment classification method. Thus, the tracking process facilitates the pedestrian detection performance.

**Figure 13:**
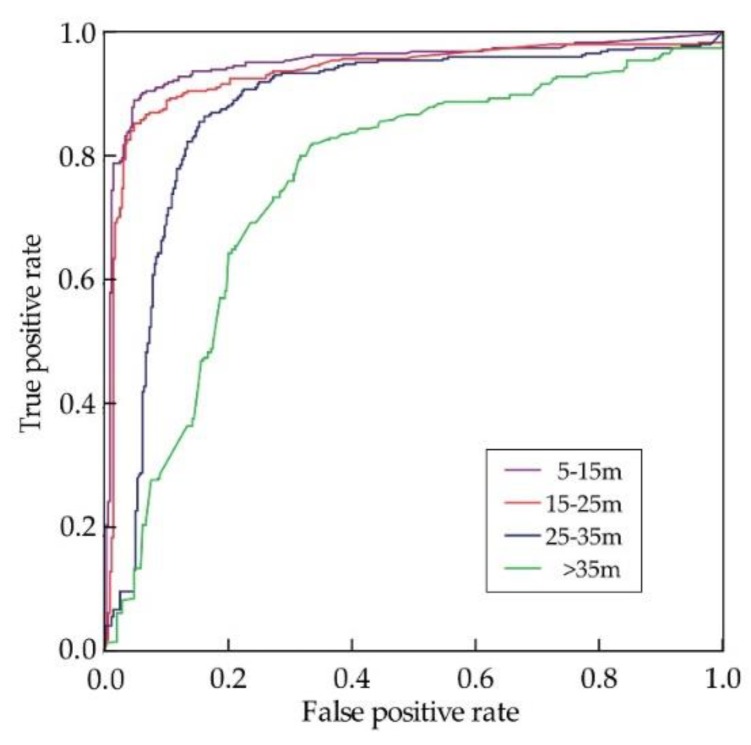
The performance of the proposed track classification algorithm based on Adaboost classifier and 18 features at various ranges from the laser scanner sensor to the pedestrian.

Due to the sparsity of the point cloud at long range, the pedestrian range affects the pedestrian detection performance. Figure 13 shows the quantitative performance analysis of the proposed track classification algorithm based on Adaboost classifier and 18 features at various ranges from the laser scanner sensor to the pedestrian. We can see that the best performance is between 5 m and 25 m, and good performance up to 35 m, beyond which the pedestrian detection performance declines significantly.

## 4. Conclusions

This study developed a novel pedestrian detection algorithm via a four-layer laser scanner. First, the raw point cloud is clustered into disjunctive segments, and three types of the features are developed including number-of-points-based, geometric and statistical features. Then, the univariate feature selection and feature correlation analysis procedures are conducted to select the effective features and remove the redundant features. Based on the segment classification, the particle filter and PDAF are combined to achieve the track classification to reduce false classification caused by the occlusion. In the road experiment, the laser scanner was mounted on an actual vehicle to collect the point cloud of surrounding environment, and the performance of the proposed pedestrian detection method is tested. Experimental results demonstrated that AdaBoost and BPNN classifiers based on 18 features have their own advantages for pedestrian detection using multi-layer laser scanner in terms of the detection performance and computation time. Moreover, the proposed pedestrian detection method based on segment and track classification using 18 features is effective even when the temporary occlusion among the pedestrians occurs.

## Figures and Tables

**Table 1:** Number-of-points-based features.

#	Expression	Description
1	*N_c_*	Number of points
2	*N_c_*_1_	Number of points from the first layer
3	*N_c_*_2_	Number of points from the second layer
4	*N_c_*_3_	Number of points from the third layer
5	*N_c_*_4_	Number of points from the fourth layer
6	*n_l_*	The number of the layers with more than two points
7	*n_k_*	Slope of the linear fit of the number of scan points vs. layers
8	*n_a_*	First order coefficient of the quadratic fit of the number of points vs. each layer
9	*n_b_*	Second order coefficient of the quadratic fit of the number of points vs. each layer
10	*D*	The distance between the central point of the horizontal projection and the origin of the coordinate system
11	*d*	The minimum distance between the horizontal projection points and the origin of the coordinate system

**Table 2:** *Geometric* features.

#	Formula	Description
12	$\sum_{i=1}^{N_{c}}{\left(p_{xy,i}-{\hat{p}}_{l,i}\right)}^{2}/N_{c}$	Linearity: ${\hat{p}}_{l,i}$ corresponds to the fitted line
13	*l*	Length along the fitted line at the horizontal plane
14	*w*	Length along the direction perpendicular to the fitted line at the horizontal plane
15	l×w	The area of the fitting rectangle at the horizontal plane
16	*N_c_*_1_ */A*_1_	The density of the points from the first layer
17	*N_c_*_2_ */A*_2_	The density of the points from the second layer
18	*N_c_*_3_ */A*_3_	The density of the points from the third layer
19	*N_c_*_4_ */A*_4_	The density of the points from the fourth layer
20	*A*_1_ + *A*_2_ + *A*_3_ + *A*_4_	Sum of the areas of four layers
21	(*A*_1_ + *A*_2_ + *A*_3_ + *A*_4_)/4	The average value of the total areas of four layers
22	$\sqrt{\Delta X^{2}+\Delta Y^{2}}$	Cartesian dimension
23	$\sum_{i=2}^{N_{c}}\left(p_{xy,i}-p_{xy,i-1}\right)$	Length of the connected curves
24	${\sum_{i=2}^{N_{c}}\left(|p_{xy,i}-p_{xy,i-1}|-\bar{d}\right)}^{2}/(N_{c}-1)$	The variance of the connected curves
25	$\sum_{i=2}^{N_{c}}\left(p_{xy,i}-p_{xy,i-1}\right)/l$	Bending of the connected curves
26	$\sum_{i=1}^{N_{c}-2}\alpha_{i}/(N_{c}-2)$	The inscribed angle mean (IAM)
27	$\sum_{i=1}^{N_{c}}{\left(\alpha -\mathrm{IAM}\right)}^{2}/(N_{c}-2)$	The inscribed angle variance
28	$\sum_{i=1}^{N_{c}}{\left(P_{xy,i}-{\hat{P}}_{a,i}\right)}^{2}/N_{c}$	Circularity: ${\hat{P}}_{a,i}$ corresponds to the fitted circle
29	*r*	The radius of the fitted circle

**Table 3:** *Statistical* features.

#	Formula	Description
30	$\sqrt{\frac{1}{N_{c}}\sum_{i=1}^{N_{c}}\left||p_{3D,i}-{\bar{p}}_{3D}|\right|}$	Standard deviation for 3D, ${\bar{p}}_{3D}$ is 3D centroid
31	$\sqrt{\frac{1}{N_{c}}\sum_{i=1}^{N_{c}}{\left(p_{xy,i}-{\bar{p}}_{2D}\right)}^{2}}$	Standard deviation for 2D, ${\bar{p}}_{2D}$ is the mean of the horizontal projection points
32	$\sum_{i=1}^{N_{c}}{\left(p_{xy,i}-D_{m}\right)}^{2}/N_{c}$	Variance for 2D, *D_m_* is the median of the horizontal projection points
33	$\sum_{i=1}^{N_{c}}{\left(p_{xy,i}-{\bar{p}}_{2D}\right)}^{2}/N_{c}$	Second central moment
34	$\sum_{i=1}^{N_{c}}{\left(p_{xy,i}-{\bar{p}}_{2D}\right)}^{3}/N_{c}$	Third central moment
35	$\sum_{i=1}^{N_{c}}{\left(p_{xy,i}-{\bar{p}}_{2D}\right)}^{4}/N_{c}$	Fourth central moment

**Table 4:** The average classification failure rate of each feature classifier.

Feature	Failure Rate	Feature	Failure Rate	Feature	Failure Rate	Feature	Failure Rate
1	23.9%	10	27.1%	19	32.8%	***28***	***44.6%***
2	34.6%	11	30.2%	20	34.4%	***29***	***41.3%***
3	26.8%	***12***	***38.0%***	21	31.1%	30	32.6%
4	34.3%	***13***	***35.5%***	***22***	***38.7%***	31	33.8%
5	30.8%	14	32.3%	***23***	***43.8%***	32	27.2%
***6***	***45.9%***	15	25.1%	***24***	***46.1%***	33	33.9%
***7***	***37.5%***	16	26.2%	25	31.4%	34	21.4%
8	34.5%	17	34.3%	26	33.0%	35	24.7%
9	33.8%	***18***	***37.1%***	27	28.8%		

**Table 5:** The statistical results of the correlation larger than 0.9.

	1	3	4	5	8	9	10	11	19	31	33	34	35
1	1.00	***0.90***	0.83	0.72	−0.09	0.01	0.60	0.59	0.01	−0.06	−0.09	−0.07	−0.04
3		1.00	0.76	0.73	−0.34	0.33	0.52	0.51	0.08	0.02	−0.01	0.00	0.01
4			1.00	***0.94***	−0.23	0.06	0.61	0.61	−0.11	−0.16	−0.21	−0.19	−0.16
5				1.00	−0.06	−0.14	0.50	0.50	−0.16	−0.22	−0.25	−0.22	−0.19
8					1.00	***−0.94***	−0.16	−0.16	0.14	0.11	0.18	0.18	0.22
9						1.00	0.13	0.13	0.00	0.05	−0.02	−0.03	−0.08
10							1.00	***1.00***	0.42	0.42	0.33	0.34	0.33
11								1.00	0.43	0.43	0.34	0.35	0.35
19									1.00	***0.91***	0.88	0.68	0.84
31										1.00	***0.97***	0.81	0.84
33											1.00	0.73	0.81
34												1.00	***0.99***
35													1.00

**Table 6:** The redundant features based on the value of correlation in descending order.

The Features with the Correlation that Exceeds the Threshold 0.9	The Correlation Coefficient	The Classification Error Rate	The Feature that Need to be Removed
Feature 10 and 11	0.998603	27.1% < 30.2%	***Feature 11***
Feature 34 and 35	0.985861	21.4% < 24.7%	***Feature 35***
Feature 31 and 33	0.968718	33.8% < 33.9%	***Feature 33***
Feature 8 and 9	−0.944697	34.5% > 33.8%	***Feature 8***
Feature 4 and 5	0.944610	34.3% > 30.8%	***Feature 4***
Feature 19 and 31	0.907311	32.8% < 33.8%	***Feature 31***
Feature 1 and 3	0.901068	23.9% < 26.8%	***Feature 3***

**Table 7:** The statistical results of pedestrian detection.

Classifier	Feature Set	Accuracy	Time/100 Samples	AUC
BPNN	35 features	81.6%	6.19 ms	0.898
	18 features	90.7%	6.03 ms	0.936
Adaboost	35 features	94.5%	13.29 ms	0.963
	18 features	93.2%	8.68 ms	0.952

**Table 8:** The results of pedestrian detection.

Method	Classifier + Feature Set	Accuracy
Segment classification	BPNN + 18 features	80.7%
	Adaboost + 18 features	85.2%
Track classification	BPNN + 18 features	93.7%
	Adaboost + 18 features	95.9%
